# Supplementary material for: Function of bidirectional sensitivity in the otolith organs established by transcription factor Emx2
Source: Nat Commun. 2022 Oct 24;13:6330. doi: 10.1038/s41467-022-33819-3 (PMC9592604; doi:10.1038/s41467-022-33819-3)
Supplement: Supplementary file 1 — Supplementary Information [file 41467_2022_33819_MOESM1_ESM.pdf]

Supplementary information

Title: Function of bidirectional sensitivity in the otolith organs established by transcription factor Emx2

Ji et al.

Supplementary figures and legends

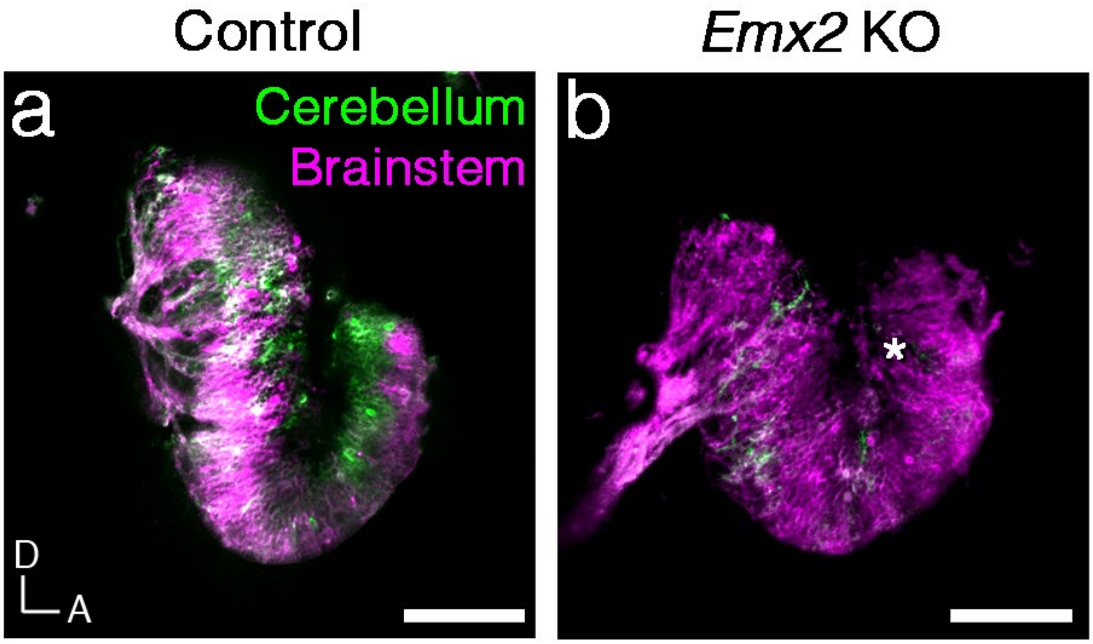

Supplementary Figure 1. Projected cerebellum afferent neurons do not reach the *Emx2* KO saccule. (a,b) Labeling of a control (a) and *Emx2* KO (b) saccule at E16.5 after neuronal tracing from the cerebellum (green) and brainstem (magenta) using NeuroVue® Maroon and NeuroVue® Red lipophilic dyes, respectively. Dye tracing from the cerebellum labels the inner region, whereas tracing from the brainstem labels the outer region of the control saccule (a). In *Emx2* KO (b), little labeling from the cerebellum is evident and labeling from the brainstem is found across the entire saccule. Asterisk indicates the inner region of *Emx2* KO saccule, which is labeled with dye from the brainstem. Control, n=3; *Emx2* KO n=3. Scale bar=100μm (a,b).

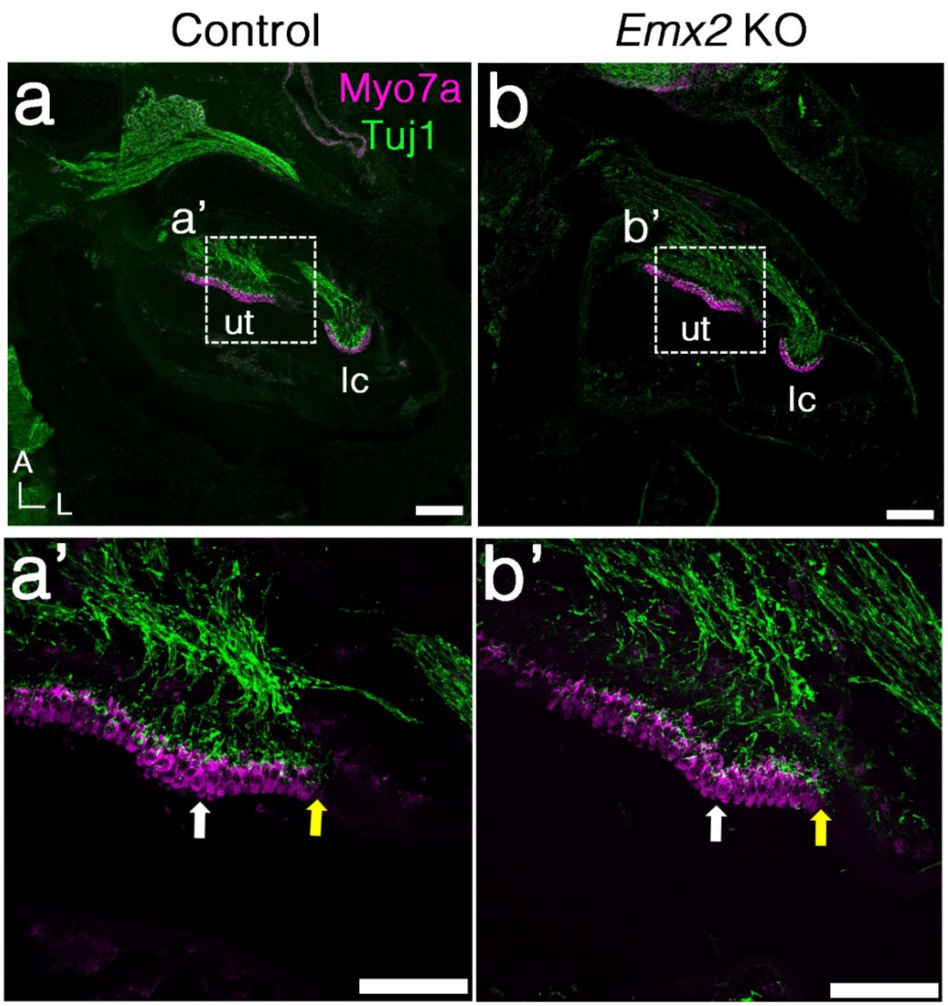

Supplementary Figure 2. Neuronal fibers are present in the lateral region of *Emx2* KO utricles. (a) Control and (b) *Emx2* KO mouse utricles stained with anti-Myo7a antibody for HCs (magenta) and anti-Tuj1 antibody for nerve fibers and neuronal cell bodies (green). Comparable nerve fibers are found beneath the lateral region (between white and yellow arrows) of both control (a’) and *Emx2* KO (b’) utricles. White arrow indicates the lateral edge of the raised striolar epithelium compared to the rest of the sensory epithelium, and the yellow arrow indicates the lateral edge of the sensory epithelium. Utricle (ut), lateral crista (lc). Control, n=5; *Emx2* KO n=4. Scale bar=200 μm in a and b, and 100 μm in a’ and b’.

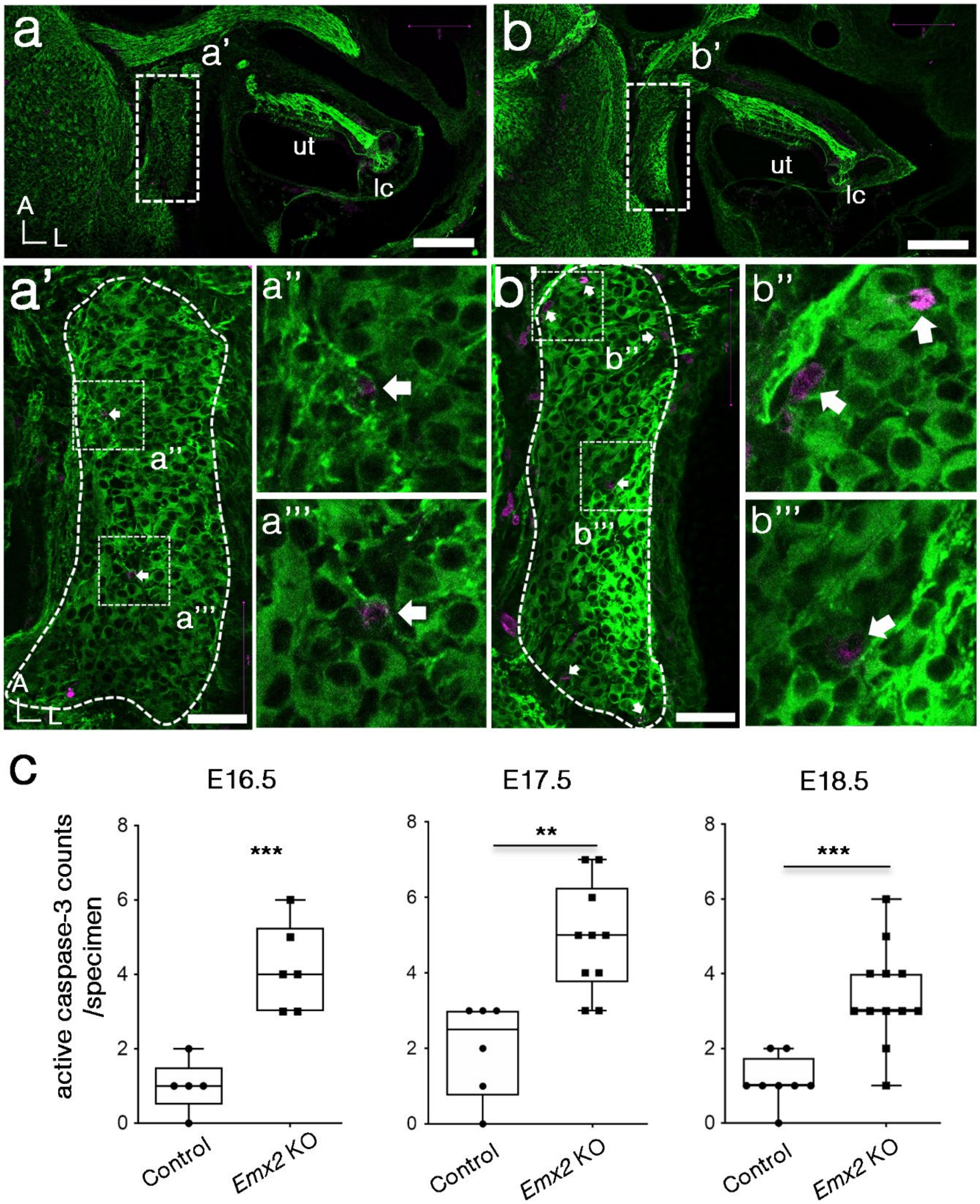

Supplementary Figure 3. Increased apoptosis in the vestibular ganglion of *Emx2* KO. Cryo-sections of controls (a - a''') and *Emx2* KO (b - b''') inner ears at E17.5, stained for apoptotic profiles using anti-active caspase 3 antibody (magenta) and

neuronal cell bodies and fibers (green) using anti-Tuj1 antibody. The vestibular ganglion (white dotted outline) shows some apoptotic profiles in controls (a’-a’”, arrow) but many more were observed in *Emx2* KO ganglion (b’-b’”). Lateral crista (lc), utricle (ut). (c) Quantification of apoptotic profiles in control and *Emx2* KO vestibular ganglia between E16.5 to E18.5. In boxplots, boxes represent the interquartile range (IQR), and the thick lines inside show the median. Whiskers denote the lowest and highest values. Unpaired t-test with two-sided, ****p* = 4.77E-04 at E16.5, ***p* = 0.0012 at E17.5, ****p* = 7.46E-05 at E18.5: control, n=5, *Emx2* KO, n=6; E17.5: control, n=6, *Emx2* KO n=10; E18.5: control, n=8, *Emx2* KO, n=12. Scale bar=500 μm in a, applies to b; 50 μm in a’, applies to b’; 25 μm in a’’, applies to b’’. Source data are provided as a Source Data file.

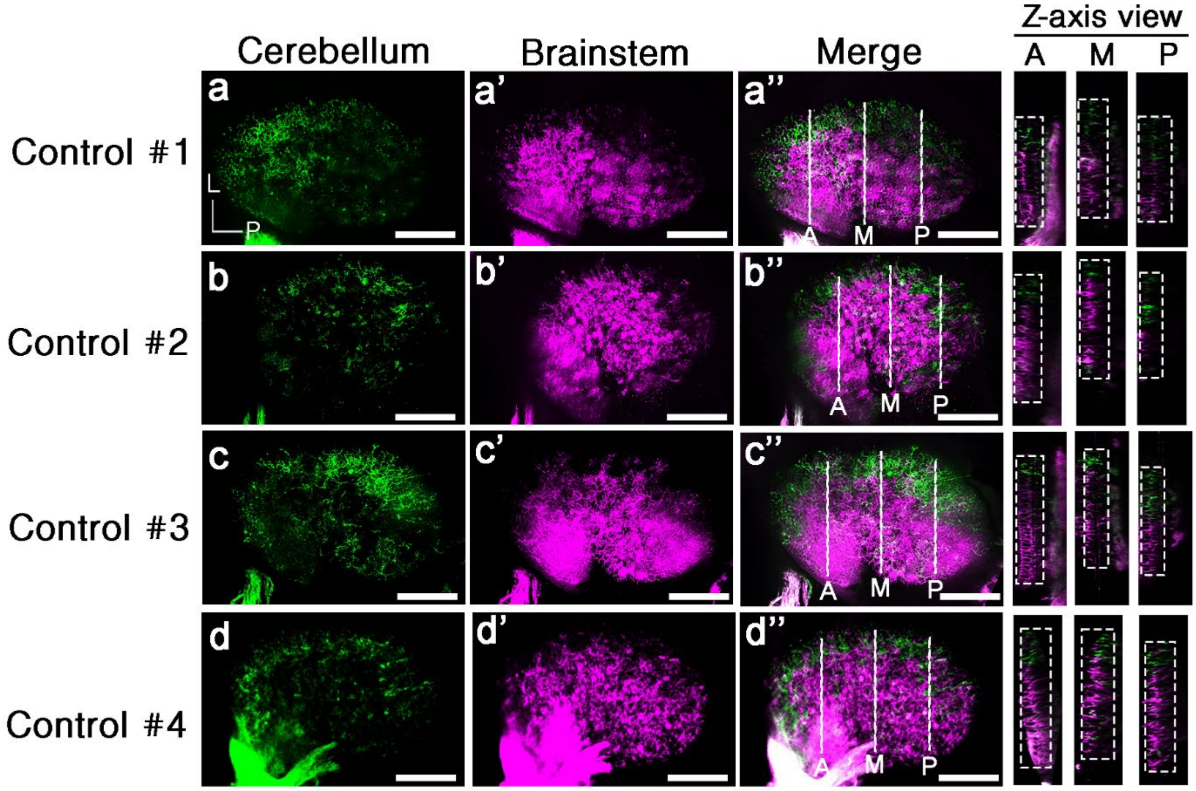

Supplementary Figure 4. Dye-tracing of the four control samples used in dye quantification. Dye-labeling in the utricle traced from the cerebellum (a-d) and brainstem (a’-d’). Z-stacked confocal images from the three selected regions, anterior, medial, and posterior (A, M, P, white dotted line, a’’-d’’) were used to quantify the dye intensity. There are four control samples to show a similar dye tracing pattern in utricle from three independent experiments. Orientation: L, lateral; P, posterior. Scale bar = 100 μm.

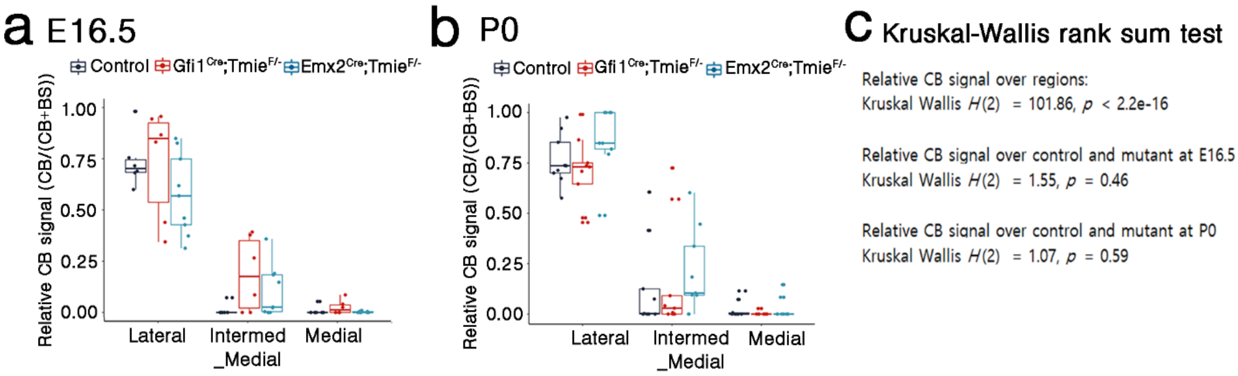

c Kruskal-Wallis rank sum test

Relative CB signal over regions:
Kruskal Wallis $H(2) = 101.86$, $p < 2.2e-16$

Relative CB signal over control and mutant at E16.5
Kruskal Wallis $H(2) = 1.55$, $p = 0.46$

Relative CB signal over control and mutant at P0
Kruskal Wallis $H(2) = 1.07$, $p = 0.59$

d Dunn's pairwise test

E16.5, overall

Comparison	Z	P.unadj	P.adj
Lateral - Intermed_Medial	4.89	1.0E-06	1.5E-06
Lateral - Medial	6.32	2.6E-10	7.8E-10
Intermed_Medial - Medial	1.43	0.15	0.15

P0, overall

Comparison	Z	P.unadj	P.adj
Lateral - Intermed_Medial	5.16	2.5E-07	3.8E-07
Lateral - Medial	7.47	7.9E-14	2.4E-13
Intermed_Medial - Medial	2.32	0.02	0.02

E16.5, Control

Comparison	Z	P.unadj	P.adj
Lateral - Intermed_Medial	3.18	1.5E-03	2.2E-03
Lateral - Medial	3.24	1.2E-03	3.6E-03
Intermed_Medial - Medial	0.06	0.95	0.95

P0, Control

Comparison	Z	P.unadj	P.adj
Lateral - Intermed_Medial	3.30	1.1E-03	1.7E-03
Lateral - Medial	4.05	5.1E-05	1.5E-04
Intermed_Medial - Medial	0.79	0.43	0.43

E16.5, *Gfi1^{Cre}; Tmie$^{F/-}$*

Comparison	Z	P.unadj	P.adj
Lateral - Intermed_Medial	2.27	0.02	0.04
Lateral - Medial	3.31	9.4E-04	2.8E-03
Intermed_Medial - Medial	1.04	0.30	0.30

P0, *Gfi1^{Cre}; Tmie$^{F/-}$*

Comparison	Z	P.unadj	P.adj
Lateral - Intermed_Medial	2.61	9.0E-03	0.01
Lateral - Medial	4.30	1.7E-05	5.0E-05
Intermed_Medial - Medial	1.69	0.09	0.09

E16.5, *Emx2^{Cre}; Tmie$^{F/-}$*

Comparison	Z	P.unadj	P.adj
Lateral - Intermed_Medial	2.83	4.7E-03	7.0E-03
Lateral - Medial	4.39	1.1E-05	3.4E-05
Intermed_Medial - Medial	1.56	0.12	0.12

P0, *Emx2^{Cre}; Tmie$^{F/-}$*

Comparison	Z	P.unadj	P.adj
Lateral - Intermed_Medial	2.82	4.8E-03	7.2E-03
Lateral - Medial	4.45	8.4E-06	2.5E-05
Intermed_Medial - Medial	1.64	0.10	0.10

Supplementary Figure 5. Quantification of dye tracing in *Tmie* mutants. (a, b) Relative cb signals in *Gfi1^{Cre}; Tmie$^{F/-}$* and *Emx2^{cre}; Tmie$^{F/-}$* (*Tmie* cKO) utricles at E16.5 (a) and P0 (b). (c, d) Kruskal-Wallis and Dunn's tests for cb signals in both E16.5 and P0. Overall, there was a significant difference between regions for any genotype in both ages (Kruskal Wallis $H(2) = 101.86$, $p < 2.2e-16$). Dunn's pairwise tests indicated the significant differences were between lateral and intermed_medial or lateral and medial (d). However, there was no significant differences in cb signals between controls and

mutants for any regions or ages (E16.5, Kruskal Wallis $H(2) = 1.55$, $p = 0.46$; P0, Kruskal Wallis $H(2) = 1.07$, $p = 0.59$). In boxplots, boxes represent the interquartile range (IQR), and the thick lines inside show the median. Whiskers denote the lowest and highest values. *Tmie*$^{F/-}$, n=2; *Gfi1*^{cre}*; Tmie*$^{F/-}$, n=2; *Emx2*^{cre}*; Tmie*$^{F/-}$, n=3 at E16.5; *Tmie*$^{F/-}$, n=3; *Gfi1*^{cre}*; Tmie*$^{F/-}$, n=3, *Emx2*^{cre}*; Tmie*$^{F/-}$, n=3 at P0. Source data are provided as a Source Data file.

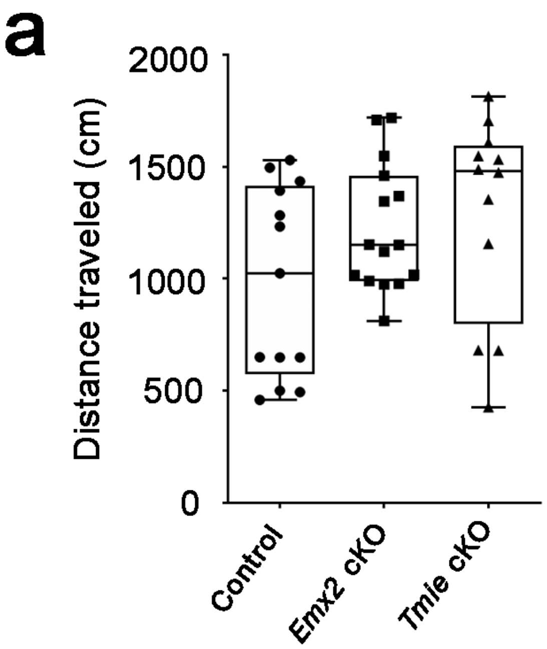

Supplementary Figure 6. Absence of hyperactivity in both *Emx2 cKO* and *Tmie cKO* mutants.

A box and whisker graph depicting the total distance traveled over a 5-minute period of control (n=13), *Emx2 cKO* (n =15) and *Tmie cKO* (n =12) mice. In boxplots, boxes represent the interquartile range (IQR), and the thick lines inside show the median. Whiskers denote the lowest and highest values. No difference between controls and mutants was observed (p = 0.1918 vs *Emx2* cKO, p = 0.1019 vs *Tmie* cKO, one-way ANOVA). Source data are provided as a Source Data file.

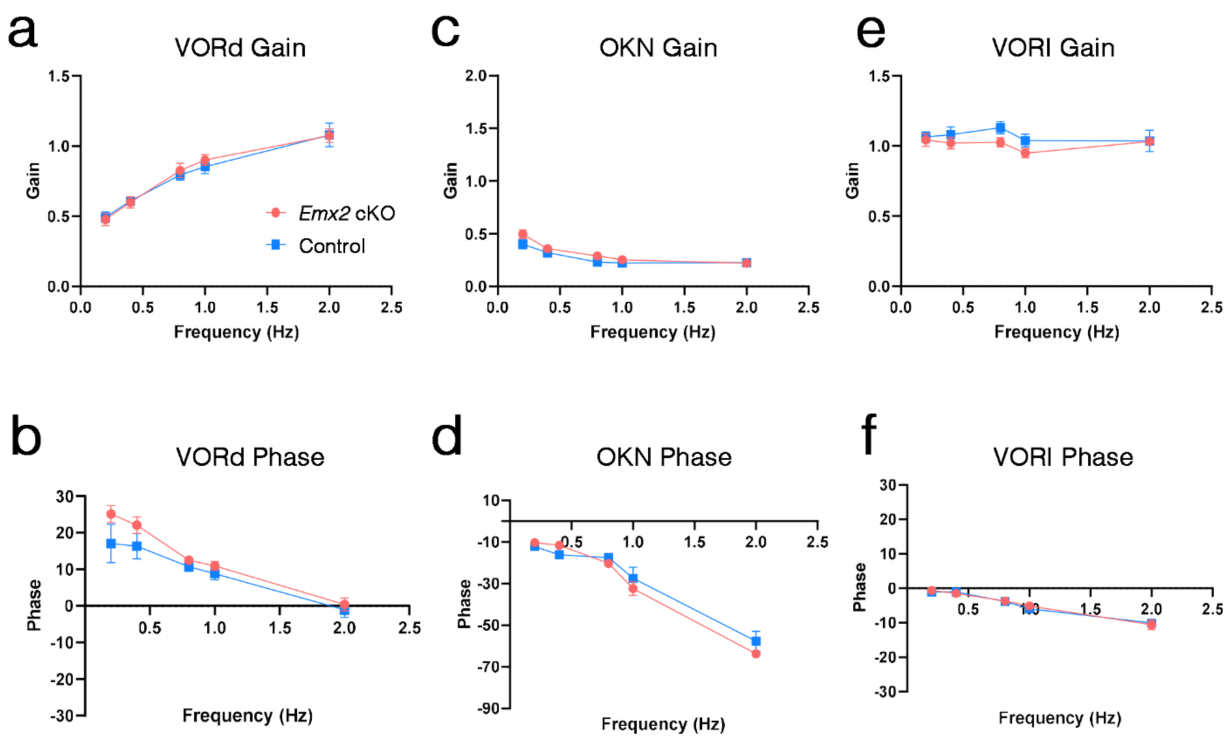

Supplementary Figure 7. VOR and OKR gains are unchanged in *Emx2* cKO. (a,b) VOR in the dark gains and phases (mean ± SEM) were comparable in *Emx2* cKO versus controls across all tested frequencies. (c,d) As a control for non-vestibular oculomotor function, we also examined the optokinetic reflex (OKR), which uses visual signals to control eye motion, allowing tracking of a moving visual scene or objects. OKR gains and phases (mean ± SEM) were comparable in *Emx2* cKO versus controls across all tested frequencies. (e,f) VOR gain was also quantified in a lit environment (referred to as VORl), where VOR functions in concert with OKR. VORl gains and phases (mean ± SEM) were comparable in *Emx2* cKO versus controls across all tested frequencies. Comparisons were two-way ANOVA with post hoc *Bonferroni*'s test with all $p > 0.05$, N = 7 vs. 4 for *Emx2* cKO vs. control, respectively. Source data are provided as a Source Data file.

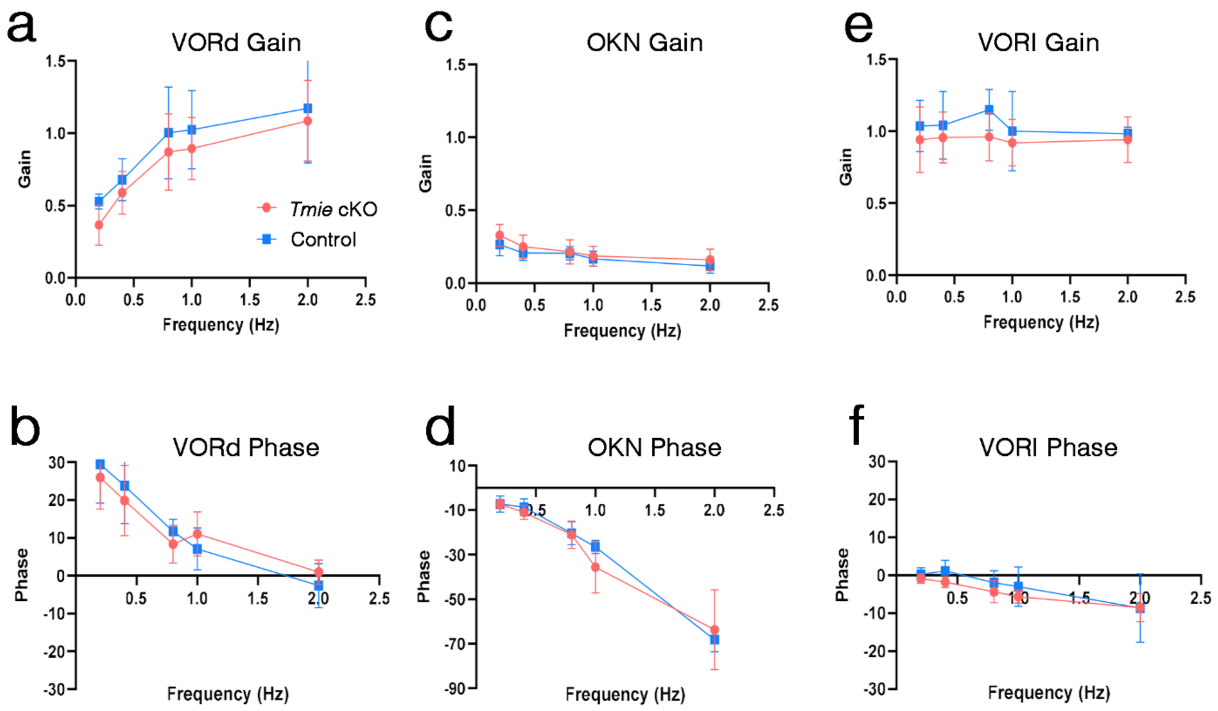

Supplementary Figure 8. VOR and OKR gains are unchanged in *Tmie* cKO. (a,b) VOR in the dark gains and phases (mean ± SEM) were comparable in *Tmie* cKO versus controls across all tested frequencies. (c,d) OKR gains and phases (mean ± SEM) were comparable in *Tmie* cKO versus controls across all tested frequencies. (e,f) VOR in the light gains and phases (mean ± SEM) were comparable in *Tmie* cKO versus controls across all tested frequencies. Comparisons were two-way ANOVA with post hoc *Bonferroni*'s test with all $p > 0.05$, N = 8 vs. 3 for *Tmie* cKO vs. control (7 *Tmie* cKO vs. 3 control for VOR in dark conditions), respectively. Source data are provided as a Source Data file.

Supplementary note 1. Statistical analysis for dye-tracing results in Figure 3

a) Multiple linear regression results for *Gfi1*^{Cre}; *Emx2*$^{F/-}$ data

Effect	*Estimate*	95% *CI*	*S.E.*	*t* value	*P* value
(Intercept)	0.72	(0.62 – 0.81)	0.048	15.01	< 2E-16
Lateral	reference				
Intermed_Medial	-0.59	(-0.73 – -0.46)	0.068	-8.79	1.5E-13
Medial	-0.67	(-0.80 – -0.53)	0.068	-9.85	1.2E-15
Control	reference				
Gfi1^{Cre}; *Emx2*$^{F/-}$	0.054	(-0.069 – 0.18)	0.062	0.87	0.38
Intermed_Medial:*Gfi1*^{Cre}; *Emx2*$^{F/-}$	0.18	(0.0076 – 0.35)	0.087	2.08	0.041
Medial:*Gfi1*^{Cre}; *Emx2*$^{F/-}$	0.031	(-0.14 – 0.20)	0.087	0.36	0.72
Observations	90				
R^2	0.76				
Adjusted R^2	0.75				
Residual Std. Error	0.17 (df = 84)				
F Statistic	53.08 (df = 5; 84)				
p value	< 2.2E-16				

b) Kruskal-Wallis test and Dunn's pairwise comparisons

Kruskal-Wallis rank sum test

Relative CB signal over Regions
Kruskal Wallis $H(2) = 57.54$, $p = 3.2E\text{-}13$

Post-hoc Dunn's pairwise test of Controls

Comparison	Z	P.unadj	P.adj
Lateral - Intermed_Medial	3.83	1.3E-04	2.6E-04
Lateral - Medial	4.45	8.5E-06	2.5E-05
Intermed_Medial - Medial	0.62	0.53	0.53

Post-hoc Dunn's pairwise test of *Gfi1*^{Cre}; *Emx2*$^{F/-}$ mutants

Comparison	Z	P.unadj	P.adj
Lateral - Intermed_Medial	3.57	3.6E-04	7.1E-04
Lateral - Medial	5.90	3.6E-09	1.1E-08
Intermed_Medial - Medial	2.33	0.02	0.02

Kruskal-Wallis rank sum test

Relative CB signal over control and mutant Lateral regions
Kruskal Wallis $H(1) = 1.21$, $p = 0.27$

Relative CB signal over control and mutant Intermed_Medial regions
Kruskal Wallis $H(1) = 9.82$, $p = 1.7E\text{-}03$

Relative CB signal over control and mutant Medial regions
Kruskal Wallis $H(1) = 2.37$, $p = 0.12$

Supplementary note 2. Statistical analysis for dye-tracing results in Figure 4

a) Multiple linear regresssion results for *Sox2*^{CreER}; *Rosa*^{Emx2} data

Effect	*Estimate*	95% *CI*	*S.E.*	*t* value	*P* value
(Intercept)	0.72	(0.62 – 0.81)	0.047	15.17	5.3E-28
Lateral	reference				
Intermed_Medial	-0.59	(-0.73 – -0.46)	0.067	-8.89	2.2E-14
Medial	-0.67	(-0.80 – -0.53)	0.067	-9.95	9.4E-17
Control	reference				
Sox2^{CreER}; *Rosa*^{Emx2} at E13.5	-0.23	(-0.36 – -0.11)	0.063	-3.65	0.00042
Sox2^{CreER}; *Rosa*^{Emx2} at E15.5	-0.27	(-0.40 – -0.14)	0.067	-4.01	0.00012
Intermed_Medial *:* *Sox2*^{CreER}; *Rosa*^{Emx2} at E13.5	0.4	(0.22 – 0.58)	0.091	4.37	3.00E-05
Medial *:* *Sox2*^{CreER}; *Rosa*^{Emx2} at E13.5	0.32	(0.14 – 0.50)	0.091	3.54	0.0006
Intermed_Medial : *Sox2*^{CreER}; *Rosa*^{Emx2} at E15.5	0.39	(0.20 – 0.58)	0.096	4.1	8.2E-05
Medial : *Sox2*^{CreER}; *Rosa*^{Emx2} at E15.5	0.39	(0.2 – 0.58)	0.095	4.1	7.1E-05
Observations	112				
R^2	0.62				
Adjusted R^2	0.59				
Residual Std. Error	0.16 (df = 103)				
F Statistic	21.1 (df = 8; 103)				
p value	< 2.2E-16				

b) Kruskal-Wallis test and Dunn's pairwise comparisons

Kruskal-Wallis rank sum test

Relative CB signal over Regions
Kruskal Wallis $H(2) = 45.40$, $p = 1.4E-10$

Post-hoc Dunn's pairwise test of Controls

Comparison	Z	P.unadj	P.adj
Lateral - Intermed_Medial	3.83	1.3E-04	2.6E-04
Lateral - Medial	4.45	8.5E-06	2.5E-05
Intermed_Medial - Medial	0.62	0.53	0.53

Post-hoc Dunn's pairwise test of *Sox2*^{CreER}; *Rosa*^{Emx2} mutants at E13.5

Comparison	Z	P.unadj	P.adj
Lateral - Intermed_Medial	1.21	0.23	0.23
Lateral - Medial	3.07	2.2E-03	6.5E-03
Intermed_Medial - Medial	1.86	0.06	0.13

Post-hoc Dunn's pairwise test of *Sox2*^{CreER}; *Rosa*^{Emx2} mutants at E15.5

Comparison	Z	P.unadj	P.adj
Lateral - Intermed_Medial	1.90	0.06	0.12
Lateral - Medial	3.28	1.1E-03	3.2E-03

Intermed_Medial - Medial	1.38	0.17	0.17

Kruskal-Wallis rank sum test

Relative CB signal over E13.5 and E15.5 Lateral regions
Kruskal Wallis $H(2) = 10.60$, $p = 5.0E-03$

Relative CB signal over E13.5 and E15.5 Intermed_Medial regions
Kruskal Wallis $H(2) = 9.60$, $p = 8.2E-03$

Relative CB signal over E13.5 and E15.5 Medial regions
Kruskal Wallis $H(2) = 6.58$, $p = 0.037$

Dunn's pairwise test

Lateral region

Comparison	Z	P.unadj	P.adj
Control - *Sox2*^{CreER}; *Rosa*^{Emx2} at E13.5	2.59	0.010	0.015
Control - *Sox2*^{CreER}; *Rosa*^{Emx2} at E15.5	3.04	0.0023	7.0E-03
Sox2^{CreER}; *Rosa*^{Emx2} at E13.5 - E15.5	0.62	0.53	0.53

Intermed_Medial region

Comparison	Z	P.unadj	P.adj
Control - *Sox2*^{CreER}; *Rosa*^{Emx2} at E13.5	-3.00	2.7E-03	8.0E-03
Control - *Sox2*^{CreER}; *Rosa*^{Emx2} at E15.5	-2.23	0.026	0.039
Sox2^{CreER}; *Rosa*^{Emx2} at E13.5 - E15.5	0.65	0.51	0.51

Medial region

Comparison	Z	P.unadj	P.adj
Control - *Sox2*^{CreER}; *Rosa*^{Emx2} at E13.5	-2.49	0.013	0.038
Control - *Sox2*^{CreER}; *Rosa*^{Emx2} at E15.5	-1.83	0.067	0.10
Sox2^{CreER}; *Rosa*^{Emx2} at E13.5 - E15.5	0.56	0.58	0.58

Supplementary note 3. Statistical analysis for dye-tracing results in Figure 5

a) Multiple linear regression results for *Gfi1*^{Cre}; *Rosa*^{Emx2} and *Plp*^{CreER}; *Rosa*^{Emx2} data

Effect	*Estimate*	95% *CI*	*S.E.*	*t* value	*P* value
(Intercept)	0.72	(0.61 – 0.83)	0.055	13.07	4.2E-28
Lateral	reference				
Intermed_Medial	-0.59	(-0.75 – -0.44)	0.078	-7.66	1.0E-12
Medial	-0.67	(-0.82 – -0.51)	0.078	-8.57	3.8E-15
Control	reference				
Gfi1^{Cre}; *Rosa*^{Emx2}	-0.084	(-0.22 – 0.048)	0.067	-1.26	0.21
Plp^{CreER}; *Rosa*^{Emx2}	-0.081	(-0.21 – 0.047)	0.065	-1.25	0.21
Intermed_Medial : *Gfi1*^{Cre}; *Rosa*^{Emx2}	0.46	(0.27 – 0.65)	0.095	4.84	2.8E-06
Medial : *Gfi1*^{Cre}; *Rosa*^{Emx2}	0.29	(0.10 – 0.48)	0.095	3.07	2.4E-03
Intermed_Medial *: Plp*^{CreER}; *Rosa*^{Emx2}	0.27	(0.090 – 0.45)	0.092	2.95	3.6E-03
Medial : Plp^{CreER}; *Rosa*^{Emx2}	0.28	(0.10 – 0.47)	0.092	3.07	2.5E-03
Observations	195				
R^2	0.55				
Adjusted R^2	0.53				
Residual Std. Error	0.19 (df = 186)				
F Statistic	27.9 (df = 8; 186)				
p value	< 2.2E-16				

b) Kruskal-Wallis test and Dunn's pairwise comparisons

Kruskal-Wallis rank sum test

Relative CB signal over Regions
Kruskal Wallis $H(2) = 74.16$, $p < 2.2e-16$

Post-hoc Dunn's pairwise test of Controls

Comparison	Z	P.unadj	P.adj
Lateral - Intermed_Medial	3.83	1.3E-04	2.6E-04
Lateral - Medial	4.45	8.5E-06	2.5E-05
Intermed_Medial - Medial	0.62	0.53	0.53

Post-hoc Dunn's pairwise test of *Gfi1*^{Cre}*; Rosa*^{Emx2} mutants

Comparison	Z	P.unadj	P.adj
Lateral - Intermed_Medial	1.70	0.09	0.09
Lateral - Medial	4.94	7.9E-07	2.4E-06
Intermed_Medial - Medial	3.23	1.2E-03	2.4E-03

Post-hoc Dunn's pairwise test of *Plp*^{CreER}*; Rosa*^{Emx2} mutants

Comparison	Z	P.unadj	P.adj
Lateral - Intermed_Medial	4.29	1.8E-05	3.5E-05
Lateral - Medial	4.96	7.1E-07	2.1E-06
Intermed_Medial - Medial	0.67	0.51	0.51

Kruskal-Wallis rank sum test

Relative CB signal over control and mutant Lateral regions
Kruskal Wallis $H(2) = 1.00$, $p = 0.61$

Relative CB signal over control and mutant Intermed_Medial regions
Kruskal Wallis $H(2) = 20.16$, $p = 4.2E-05$

Relative CB signal over control and mutant Medial regions
Kruskal Wallis $H(2) = 14.13$, $p = 8.6E-04$

Dunn's pairwise test

Lateral region

Comparison	Z	P.unadj	P.adj
Control - *Gfi1*^{Cre}; *Rosa*^{Emx2}	0.90	0.37	0.55
Control - *Plp*^{CreER}; *Rosa*^{Emx2}	0.93	0.35	1.00
Gfi1^{Cre}; *Rosa*^{Emx2} - *Plp*^{CreER}; *Rosa*^{Emx2}	-0.0048	1.00	1.00

Intermed_Medial region

Comparison	Z	P.unadj	P.adj
Control - *Gfi1*^{Cre}; *Rosa*^{Emx2}	-4.45	8.5E-06	2.6E-05
Control - *Plp*^{CreER}; *Rosa*^{Emx2}	-2.65	0.008	0.012
Gfi1^{Cre}; *Rosa*^{Emx2} - *Plp*^{CreER}; *Rosa*^{Emx2}	2.44	0.015	0.015

Medial region

Comparison	Z	P.unadj	P.adj
Control - *Gfi1*^{Cre}; *Rosa*^{Emx2}	-3.37	7.5E-04	1.1E-03
Control - *Plp*^{CreER}; *Rosa*^{Emx2}	-3.53	4.2E-04	1.3E-03
Gfi1^{Cre}; *Rosa*^{Emx2} - *Plp*^{CreER}; *Rosa*^{Emx2}	-0.05	0.96	0.96
