# Supplementary material for: Function of bidirectional sensitivity in the otolith organs established by transcription factor Emx2
Source: Nat Commun. 2022 Oct 24;13:6330. doi: 10.1038/s41467-022-33819-3 (PMC9592604; doi:10.1038/s41467-022-33819-3)
Supplement: Supplementary file 3 — Reporting Summary [file 41467_2022_33819_MOESM3_ESM.pdf]

nature research

Corresponding author(s): Doris Wu

Last updated by author(s): Sep 22, 2022

Reporting Summary

Nature Research wishes to improve the reproducibility of the work that we publish. This form provides structure for consistency and transparency in reporting. For further information on Nature Research policies, see Authors & Referees and the Editorial Policy Checklist .

Statistics

For all statistical analyses, confirm that the following items are present in the figure legend, table legend, main text, or Methods section.

n/a	Confirmed	
☐	☒	The exact sample size (*n*) for each experimental group/condition, given as a discrete number and unit of measurement
☐	☒	A statement on whether measurements were taken from distinct samples or whether the same sample was measured repeatedly
☐	☒	The statistical test(s) used AND whether they are one- or two-sided<br>*Only common tests should be described solely by name; describe more complex techniques in the Methods section.*
☒	☐	A description of all covariates tested
☐	☒	A description of any assumptions or corrections, such as tests of normality and adjustment for multiple comparisons
☐	☒	A full description of the statistical parameters including central tendency (e.g. means) or other basic estimates (e.g. regression coefficient) AND variation (e.g. standard deviation) or associated estimates of uncertainty (e.g. confidence intervals)
☐	☒	For null hypothesis testing, the test statistic (e.g. *F*, *t*, *r*) with confidence intervals, effect sizes, degrees of freedom and *P* value noted<br>*Give P values as exact values whenever suitable.*
☒	☐	For Bayesian analysis, information on the choice of priors and Markov chain Monte Carlo settings
☒	☐	For hierarchical and complex designs, identification of the appropriate level for tests and full reporting of outcomes
☒	☐	Estimates of effect sizes (e.g. Cohen's *d*, Pearson's *r*), indicating how they were calculated

Our web collection on statistics for biologists contains articles on many of the points above.

Software and code

Policy information about availability of computer code

Data collection	Scanning confocal microscope: LSM780, Upright microscope: ZEN (2012), VsEP (BioSig), aVOR: LabVIEW 2020, Rotarod: ROTAROD
Data analysis	ImageJ (1.52h), Prism (7), Topscan (3.0), ForcedSwimScan™ (2.0), R (version 4.1.3.), G*Power (version 3.1.9.6)

For manuscripts utilizing custom algorithms or software that are central to the research but not yet described in published literature, software must be made available to editors/reviewers. We strongly encourage code deposition in a community repository (e.g. GitHub). See the Nature Research guidelines for submitting code & software for further information.

Data

Policy information about availability of data

All manuscripts must include a data availability statement. This statement should provide the following information, where applicable:

- Accession codes, unique identifiers, or web links for publicly available datasets
- A list of figures that have associated raw data
- A description of any restrictions on data availability

All relevant data are included in this article and its Supplementary Information files. Source data are provided with this paper.

Field-specific reporting

Please select the one below that is the best fit for your research. If you are not sure, read the appropriate sections before making your selection.

☒ Life sciences ☐ Behavioural & social sciences ☐ Ecological, evolutionary & environmental sciences

Life sciences study design

All studies must disclose on these points even when the disclosure is negative.

Sample size	A minimum of three separate collections of single cells were made for each time point. Sample size was described in Methods section, the main text or figure legend, if not already plotted on individual graphs in the figures. All quantifications are results from at least three samples, unless indicated otherwise.
Data exclusions	Others excluded for dye tracing experiments were described in Methods 'Outliers were identified with the diagnostics plots. The outlier exclusion was on two-fold basis: First, the outliers need to lie outside 1.5*IQR (Inter Quartile Range), as visualized in the box-whisker plot. Second, the outliers were influential datapoints identified by Cook's distance, which were at least greater than four times the mean. '.
Replication	Replications are indicated in the Methods section and figure legend of each graph. All in situ hybridization results were reproducible and conducted at least twice.
Randomization	Pregnant females of the appropriate genotype were randomly selected and then collected for dye tracing. All mice for behavior were randomly selected based on the litters a collected as they become available.
Blinding	During data collection for dye tracing, investigators were not blinded, because we performed dye tracing after genotyping of embryo samples. But after data collection, it was blinded for analysis of dye intensity. However, data collection and analyses of behavioral tests of mice were blinded.

Reporting for specific materials, systems and methods

We require information from authors about some types of materials, experimental systems and methods used in many studies. Here, indicate whether each material, system or method listed is relevant to your study. If you are not sure if a list item applies to your research, read the appropriate section before selecting a response.

Materials & experimental systems

n/a	Involved in the study
☐	☒ Antibodies
☒	☐ Eukaryotic cell lines
☒	☐ Palaeontology
☐	☒ Animals and other organisms
☒	☐ Human research participants
☒	☐ Clinical data

Methods

n/a	Involved in the study
☒	☐ ChIP-seq
☒	☐ Flow cytometry
☒	☐ MRI-based neuroimaging

Antibodies

Antibodies used	Primary antibodies of anti-βII spectrin (1:500; Cat #: 612562, BD Biosciences, San Jose, CA) and rat anti-tdTomato 16D7 (1:200; Cat #: EST203, Kerafast, Boston, MA), mouse anti- beta-III tubulin (Tuj1) at 1:500 dilution (Cat #: MAB1195, R&D systems, Minneapolis, MN), rabbit anti-cleaved caspase-3 D175 at 1:300 dilution (Cat #: 9661, Cell signaling, Danvers, MA) and rabbit polyclonal anti-Myosin7a at 1:1000 dilution (Cat #:<br>25-6790, Proteus Bioscience, Ramona, CA),<br>secondary antibodies (Goat anti-mouse IgG Alexa Fluor 488, Cat #: A11029 and Goat anti-rat IgG Alexa Flour 568, Cat #: A11077, Goat anti-rabbit IgG Alexa Flour 568, Cat #: A21069, Thermo Fisher Scientific, Waltham, MA) at 1:1000 dilution
Validation	βII spectrin (https://www.bdbiosciences.com/en-fr/products/reagents/microscopy-imaging-reagents/immunofluorescence-reagents/purified-mouse-anti-spectrin-ii.612562), tdTomato 16D7 (https://www.kerafast.com/item/803/anti-tdtomato-16d7-antibody), beta-III tubulin (Tuj1) (https://www.rndsystems.com/products/neuron-specific-beta-iii-tubulin-antibody-tuj-1_mab1195), cleaved caspase-3D175 (https://www.cellsignal.com/products/primary-antibodies/cleaved-caspase-3-asp175-antibody/9661), Myosin7a (https://www.labome.com/product/Proteus-Biosciences/25-6790.html)

Animals and other organisms

,Policy information about studies involving animals; ARRIVE guidelines recommended for reporting animal research

Laboratory animals	The following mouse strains were used in the study: Emx2Cre (CD-1 background) from Shinichi Aizawa at RIKEN Center (RRID:IMSR_RBRC02272); Emx2flox (Emx2F) (C57BL/6J/129 background) from Andreas Zembrzycki at the Salk Institute; Emx2+/- (mixed C57BL/6J and CD1 background) from Peter Gruss at the Max-Planck Institute (RRID:IMSR_EM:00065); Gfi1Cre (CD-1 background) from Lin Gan at Augusta University(PRID:MGI:4430258)22; Gfapcre (RRID: IRSR_JAX:004600), PlpCreER (RRID: IMSR_JAX:005975) and Rosa26tdTomato (RRID:IMSR_JAX:007914) from Jackson Laboratory; Sox2CreER from Konrad Hochedlinger at Harvard University (RRID:IMSR_JAX:017593), and Tmie+/- and Tmieflox (TmieF)(C57BL6J/129 background)from Ulrich Müller at Johns Hopkins University. We used adult mice from 4 to 10 months and utricle and saccule sample from E16.5 and P0 with both female and male.
Wild animals	not used
Field-collected samples	not used
Ethics oversight	All animal experiments were conducted under the approved NIH animal protocols at the NIH, University of Nebraska -Lincoln, Johns Hopkins University and according to NIH animal user guidelines.

Note that full information on the approval of the study protocol must also be provided in the manuscript.
